# Design and Properties of Natural Rosin-Based Phosphoester Functional Surfactants

**DOI:** 10.3390/molecules28073091

**Published:** 2023-03-30

**Authors:** Maogong Wang, Xiaofang Yang, Bing Han, Shifeng Zhang, Chunrui Han, Changlei Xia

**Affiliations:** 1 CNPC Engineering Technology R&D Company Limited, Beijing 102206, China; 2 MOE Engineering Research Center of Forestry Biomass Materials and Energy, Ministry of Education, Beijing Forestry University, Beijing 100083, China; 3 College of Materials Science and Engineering, Nanjing Forestry University, Nanjing 210037, China

**Keywords:** dehydroabietic acid, phosphate, surfactant, natural rosin

## Abstract

As an important forestry biomass resource, rosin has a wide range of applications in medicine, adhesives, surfactants and other fields. Using natural dehydroabietic acid as a raw material, dehydroabietic acid-based phosphorus monoester (DPM) and diester (DPD) surfactants were designed and synthesized. The chemical structures and self-assembly properties were characterized by FT-IR, NMR and TEM, and the effects of pH on critical micelle concentration, γ_CMC_, emulsifying properties, foam properties and micelle morphology were studied. The results showed that the CMC, γ_CMC_ value and aggregate morphology had certain pH responsiveness. The γ_CMC_ value under acidic conditions was smaller than γ_CMC_ under alkaline conditions, and the foaming performance and foam stability under acidic conditions were better than those under alkaline conditions. TEM micelle morphology studies have shown that DPM and DPD surfactants can self-assemble into rod-shaped and spherical micelle morphologies with a pH change in an aqueous solution. At the same pH, the foaming and emulsification properties of DPD were better than those of DPM. The best foaming and emulsification ability of DPD were 11.8 mL and 175 s, respectively. At the same time, the foaming ability of DPD is also affected by pH. DPD has excellent foaming properties in acidic conditions, but these disappeared in neutral conditions.

## 1. Introduction

Rosin is an important biomass resource. Developing deep processing and utilization of rosin is very important. The main component of rosin is resin acid, which is composed of a strong lipophilic ternary phenanthrene skeleton structure and a weak hydrophilic carboxyl group, and its surface activity is weak. However, resin acids have reactive groups, double bonds, carboxyl groups and other functional groups [1]. Additionally, rosin-based surfactants with excellent properties can be obtained by chemical modification. By introducing amino, ester, azo and sulfur bonds and other groups into rosin resin acid, functional surfactants with pH responsiveness, decomposability, light response and redox response can be obtained, which can be used in new applications, such as anticancer drug carriers and functional materials. By introducing an amide group in dehydroabietic acid, gemini surfactants for the preparation of mesoporous materials can be obtained [2]. However, rosin-based derivatives that serve as templates are often removed during subsequent processing. Additionally, the synthesized functional surfactants still have problems such as unstable micelles, uncontrollable self-assembly forms and pollution [3,4].

The pH-responsive surfactants have attracted the attention of researchers due to their simple preparation, reversible and controllable performance and economic and environmental protection, and have been widely used in targeted drug loading, such as SDN, adsorption and catalysis [5]. Under the action of acid or alkali, surfactants containing pH-responsive groups such as carboxyl groups, phosphate groups and amine groups all exhibit a change in hydrophilic lipophilic equilibrium value (HLB). The change of HLB value can cause the destruction and reconstruction of the micelle structure of the system, so as to realize the controllable change in the self-assembly morphology and viscosity of the system. Under acidic conditions, the tertiary amine functional group undergoes amine protonation, so the charge density of the surfactants and the hydrophilicity of the group increase [6]. In our research group, a pH-responsive tertiary amine salt surfactant (RETA) of maleic rosin was synthesized by introducing amine groups into rosin resin acid [7]. RETA has excellent surface properties and low toxicity, and can be used as the carrier of the targeted anticancer drug doxorubicin hydrochloride (DOX) [7]. Tian Chao of our research group introduced tertiary amine and ester groups into natural rosin to prepare a rosin-based cationic surfactant (RETAS). RETAS has excellent emulsifying properties, emulsion stability and pH-responsive targeted release [8]. However, micelles as drug carriers have unstable factors, such as drug-burst release.

Phosphate surfactants containing pH response groups not only have the characteristics of degradability of ester surfactants, but also have excellent wettability, emulsification and compatibility; and they are widely used in daily chemicals and other fields [9,10]. On this basis, Li Juan of our research group introduced phosphate group into rosin resin acid and synthesized a rosin-based phosphate diester surfactant (DDPDS) with dual functions of pH and decomposition [11]. DDPDS not only has excellent surface and emulsifying ability, but also uses DDPDS as an organic phosphorus source to obtain hydroxyapatite (HAP)/rosin hybrid functional materials with controllable morphology through in situ reaction [12]. The Ca-Rosin complex produced by an in-situ reaction combines rosin organic/HAP inorganic materials’ stably. The rosin terpene structure in hybrid materials greatly improves DOX’s drug-carrying capacity and also has an outstanding targeting effect.

**Figure 1:**
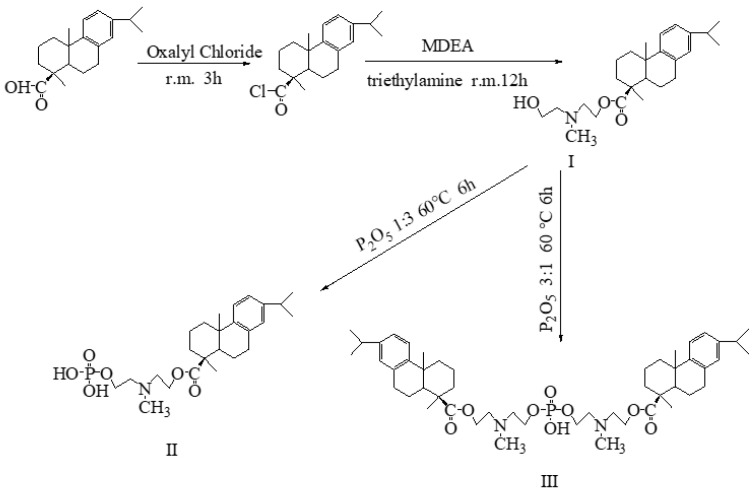
Synthetic routes of I (DDAM), II (DPM) and III (DPD).

In this study, two new surfactants were obtained by introducing two functional groups of amine and phosphate into rosin resin acid using dehydroabietic acid as raw material, and the synthesis route is shown in Figure 1. By analyzing the influences of pH, monoester phosphate and diester on foaming and emulsifying properties in detail, multifunctional rosin biomass resource-based surfactants were developed, which are expected to be used in daily necessities such as drug carriers and cosmetics.

## 2. Results and Discussion

### 2.1. Chemical Structure

**Figure 2:**
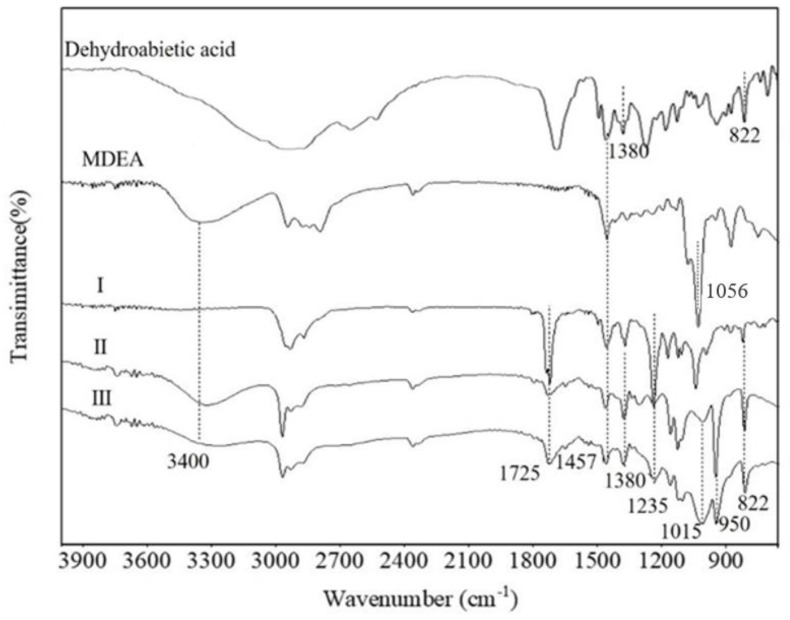
FT−IR of raw materials and productions I (DDAM), II (DPM) and III (DPD).

FT−IR study was carried out to confirm the structures of DPM and DPD. Figure 2 shows the infrared spectra of raw materials and products I (DDAM), II (DPM) and III (DPD). The absorption peaks at the 3400 and 1457 cm^−^^1^ bands are the stretching vibration peaks of –OH and methyl, respectively [13]. Compared with MDEA, compound I has new characteristic absorption peaks at 822, 1380, 1056 and 1725 cm^−^^1^. The 822 and 1380 cm^−^^1^ peaks belong to the vibration absorption peaks of the terpene structure of dehydroabietic acid [14], 1056 cm^−^^1^ belongs to the characteristic absorption peak of –OH and 1725 cm^−^^1^ belongs to the vibration peaks of the ester bond. The results show that DDAM was successfully synthesized by the esterification reaction. Unlike DDAM, both compound II and compound III had the characteristic absorption peaks of rosin (1380 and 822 cm^−^^1^), indicating that both compounds contained rosin functional groups. At the same time, the absorption peak (–P=O) at the 1235 cm^−^^1^ band and the absorption peak (–P–O–C) at the 1015 cm^−^^1^ band proved that MDEA was successfully modified by phosphorus pentoxide to synthesize phosphate esters [15]. Since DPD is a symmetric rosin-based phosphodiester, the characteristic absorption peaks of the two ester carbonyl groups in the compound structure were both at 1725 cm^−^^1^. It can be roughly judged from the absorption intensity of −OH that compound II, with high absorption intensity, was a monoester (DPM), and compound III, with weaker absorption intensity, was a diester (DPD).

### 2.2. NMR Analysis

#### 2.2.1. NMR of DPM

In Supplemental Material, Figure S1 shows the carbon nuclear magnetic spectrum of DPM. Each carbon atom’s position (^13^C NMR400 MHz, deuterated chloroform as solvent) in δ/ppm: 193.557 (C-1), 158.300 (C-2), 157.383 (C-3), 143.083 (C-4), 133.713 (C-5), 132.474 (C-6), 130.120 (C-7), 71.710 (C-8), 71.445 (C-9), 71.180 (C-10), 55.447 (C-11), 49.036 (C-12), 44.231 (C-13), 43.840 (C-14), 36.415 (C-15), 34.624 (C-16), 33.102 (C-17), 31.334 (C-18), 31.082 (C-19), 27.424 (C-20), 25.572 (C-21), 22.385 (C-22), 21.274 (C-23) and 19.924 (C-24). Among them, 193.557 is the chemical shift of ester bond C, and the signal peaks at 158.300, 157.383, 143.083, 133.713, 132.474 and 130.120 ppm are the signals of six carbon atoms in the benzene ring.

In Supplemental Material, Figure S2 shows the chemical shifts of all H elements in DPM. The main signals of 6.79–7.31 and −0.683–2.62 ppm are attributed to the rigid structure of the tricyclic skeleton of rosin [12], which further shows that DPM contains rosin functional groups.

#### 2.2.2. NMR of DPD

In the Supplemental Material, Figure S3 shows the carbon nuclear magnetic spectrum of DPD. Each carbon atom’s position (^13^C NMR400 MHz, deuterated chloroform as solvent) δ/ppm: 193.557 (C-1), 158.217 (C-2), 157.379 (C-3), 146.910 (C-4), 142.952 (C-5), 133.716 (C-6), 130.126 (C-7), 71.726 (C-8), 71.462 (C-9), 71.196 (C-10), 55.459 (C-11), 48.910 (C-12), 45.329 (C-13), 44.186 (C-14), 36.422 (C-15), 34.899 (C-16), 34.621 (C-17), 33.110 (C-18), 31.339 (C-19), 27.362 (C-20), 25.572 (C-21), 22.390 (C-22), 21.277 (C-23) and 19.937 (C-24). Among them, 193.557 is the chemical shift of the ester bond C, and the signal peaks at 158.217, 157.379, 146.910, 142.952, 133.716 and 130.126 ppm are the signals of six C atoms in the benzene ring.

In the Supplemental Material, Figure S4 shows the chemical shifts of all H elements in DPD. The main signals of 6.80–7.28 and −0.695 to 2.62 ppm are attributed to the tricyclic rigid structure of rosin [12], which further shows that DPD contains rosin functional groups.

### 2.3. Influence of Synthesis Conditions

In the synthesis, the feed ratio of n(P_2_O_5_)/n(DDAM) (1:3 and 3:1) and the reaction temperature (60, 70 and 80 °C) were investigated. The specific synthesis conditions are shown in Table 1. The ^31^P spectrum was used to identify whether DDAM had a chemical reaction with phosphorus pentoxide, and according to the sensitivity of the chemical shift of the P element in phosphoric acid and phosphorus monoester and diester to pH, the precise discrimination of phosphorus monoester and diester.

Samples 1 and 5 have phosphorous signals in Figure S5, and samples 2, 3 and 4 have no phosphorous signals, indicating that samples 1 and 5 can successfully synthesize phosphorous esters. Sample 5 may have been composed of phosphoric acid, phosphate monoester and mixture of phosphate diesters. According to the reference [16], it can be determined that the chemical shifts of 0.271, −0.683 and −1.213 ppm represent free phosphoric acid, phosphoric acid monoester and phosphoric acid diester, respectively. In order to further characterize the phosphorous monoester and phosphorous diester, the pH of the deuterated chloroform solution of the products of samples 1 and 5 was adjusted to be strongly alkaline, and the supernatant was taken for a nuclear magnetic test.

The ^31^P spectra of samples 1 and 5 are shown in Figures S6 and S7 in the attachment. The data show that the P chemical shift of sample 1 basically does not change with the changes in pH. Compared with Figure S5, the P chemical shift of sample 5 largely shifts to the left as the pH increases. According to the reference [17], it can be confirmed that sample 1 is DPD, sample 5 is DPM. The phosphoric ester was partially hydrolyzed under alkaline conditions, protonated into free phosphoric acid [18], and then reacted with sodium hydroxide to neutralize the acid and alkali; and the deprotonation formed Na_3_PO_4_ (11.477), Na_2_HPO_4_ (10.492) and NaH_2_PO_4_ (8.669).

### 2.4. Surface Properties

In the preliminary research of the laboratory, rosin-based phosphate esters were used to prepare rosin/apatite hybrid materials. The roles of rosin-based phosphate esters: one is to slowly release phosphate through hydrolysis as a phosphorus source, and the other is to form different self-assembly shape and provide a template for material formation. Preliminary research found that the pH range required for the preparation of hybrid materials is approximately between 3 and 9 [11,12], so we choose 1.42, 3.04, 6.04, 9.06 and 10.08 as the pH levels. However, the accuracy of pH values is inevitably affected by the error of the measuring instrument.

**Figure 3:**
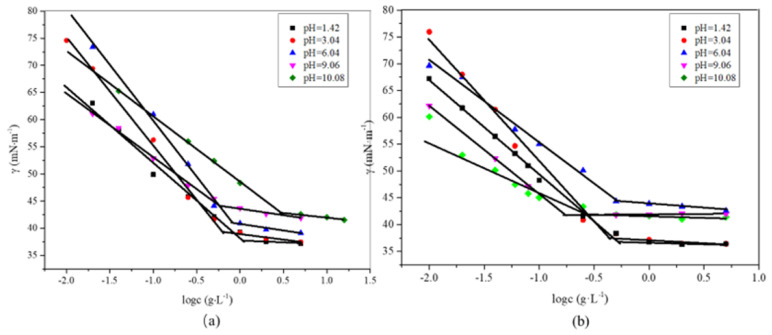
γ−logc curves of DPM (**a**) and DPD (**b**) at different pH values.

The γ−logc curves of DPM (a) and DPD (b) under five different pH conditions are shown in Figure 3, and the measurement results were affected by temperature and equipment accuracy. We calculated the critical micelle concentrations (CMCs) and the surface tension values (γ_CMC_) of DPM and DPD under the five pH values, as shown in Table 2. When pH > 6.04 or pH < 6.04, the CMC first decreases and then increases. This is because under acidic conditions, the repulsive force between the polar head groups is weakened [19], and when the pH value is further reduced, DPM is all converted into cationic surfactants containing quaternary-ammonium-salt functional groups. The repulsive force increases. Under alkaline conditions, the repulsive force between the polar head groups is weakened at the beginning. When the pH value further increases, DPM is all converted to an anionic surfactant containing phosphate functional groups, and the repulsive force between the polar head groups increases [19,20]. It can be seen in the above results that the γ_CMC_ and CMCs of DPM and DPD are both affected by pH, and the γ_CMC_ under acidic conditions is smaller than that under alkaline conditions. The change rule of the CMC of DPD is basically the same as that of DPM, but the CMC of DPD is generally lower than that of DPM. The smaller the CMC, the stronger the ability to form micelles. Compared with DPM, DPD can accumulate at a lower concentration and has a stronger aggregation ability. Due to the existence of rigid rosin acid groups, strong intermolecular hydrophobic interactions are produced, giving the surfactant DPD strong aggregation ability. In addition, the surface tension of DPD decreases first and then increases with the changing of pH. When the pH is 6.04, the γ_CMC_ and CMCs of DPM and DPD reached their maxima, which were 40.987 and 44.379 mN·m^−1^ and 1.657 and 0.612 mmol·L^−1^, respectively. In addition, the γ_CMC_ and CMCs of RETAS and SDBS were 43.09 and 43.24 mN·m^−1^ and 0.56 and 1.25 mmol·L^−1^ at 25 °C, respectively. In contrast, DPM and DPD have slightly superior surface properties [8].

### 2.5. Self-Assembly Behavior of Micelles in an Aqueous Solution

**Figure 4:**
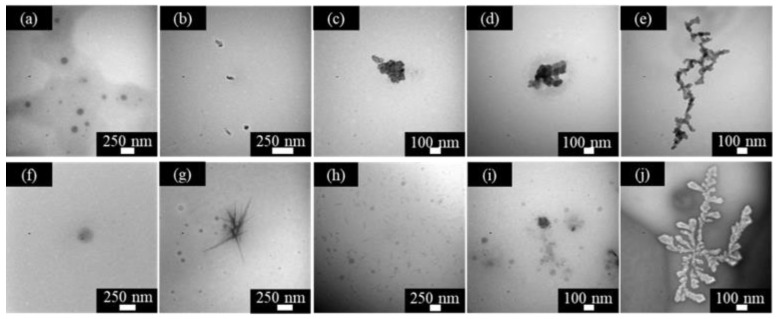
TEM imagines of DPM at different concentration and pH values—pH = 1.42: (**a**) 2 CMC and (**f**) 5 CMC; pH = 3.04: (**b**) 2 CMC and (**g**) 5 CMC; pH = 6.04: (**c**) 2 CMC and (**h**) 5 CMC; pH = 9.06: (**d**) 2 CMC and (**i**) 5CMC; pH = 10.08: (**e**) 2 CMC and (**j**) 5CMC.

pH-responsive micelles [21] refer to micellar systems containing pH-responsive groups (such as carboxyl groups, phosphoric acid groups, phenolic hydroxyl groups, or amine groups). By interacting with H^+^ or OH^−^, the hydrophilic–lipophilic balance value of the amphiphilic compound molecules in the system will change, causing the destruction and reconstruction of the micellar structure of the system, thereby achieving controllable changes in system viscosity and other properties. Figure 4 shows the morphologies of DPM and DPD at different concentrations and in different pH conditions. It can be seen in the comparison that the concentration has little effect on the structure of DPM aggregates. After increasing the concentration, the contact between the molecules is greater. Although the size changes, the original structure is basically maintained. Comparing the first row of pictures, it can be seen that the aggregates changed from spherical (Figure 4a) to rod-shaped (Figure 4b), and finally into spherical (Figure 4c–e).

The shape of an aggregate can be quantitatively described using a surfactant packing parameter defined as C_PP_ = V/(A × L). C_PP_ is related to the volume of the hydrocarbon chain (V), the effective hydrophilic head group area (A) and the chain length of the hydrophobic tail of surfactants (L) [22]. Surfactants with small C_PP_ (≤1/3) prefer to form spherical aggregates, whereas rod-like aggregates are formed with values between 1/3 and 1/2. Vesicles or lamellar like aggregates are found to form with C_PP_ values between 1/2 and 1, whereas reverse micelles or microemulsions are formed with C_PP_ > 1 [23]. As the compound contains pH-responsive groups such as tertiary amines and hydroxyl groups, we can control the effective hydrophilic head group area (A) by controlling the pH value.

**Figure 5:**
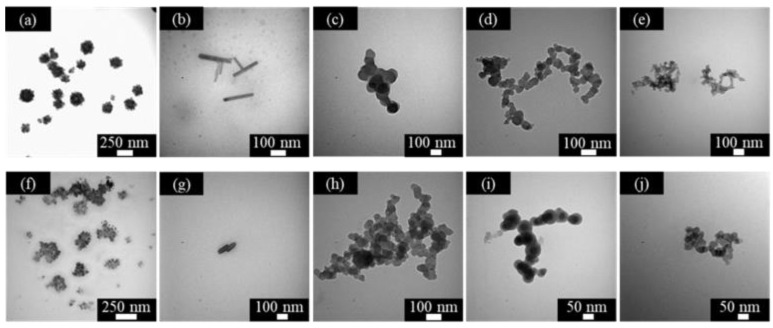
TEM images of DPD at different concentrations and pH values—pH = 1.42: (**a**) 2 CMC and (**f**) 5 CMC; pH = 3.04: (**b**) 2 CMC and (**g**) 5 CMC; pH = 6.04: (**c**) 2 CMC and (**h**) 5 CMC; pH = 9.06: (**d**) 2 CMC and (**i**) 5CMC; pH = 10.08: (**e**) 2 CMC and (**j**) 5CMC.

As the pH increases from 1.42 to 3.04, the protonated amine group is deprotonated, resulting in a decrease in “A” and an increase in C_PP_. At this time, the hydrophobic effect is greater than the repulsive force between the surfactant molecules, so the spherical micelles are transformed into rod-shaped micelles (Figure 5b). When the pH is further increased, the repulsive force between molecules increases, the value of “A” increases, and the C_PP_ decreases, thereby changing the micelles from rod-shaped to spherical. It can be seen in Figure 4j that when the concentration is five times the CMC and pH = 10.08, the surfactant molecules form a dendritic morphology. This is because DPM contains tertiary amine polar groups. The tertiary amine groups are connected to the rosin functional group with extremely strong hydrophobic properties. At high concentrations, the DPM micelles are arranged in an orderly manner, forming a structure similar to dendrites (Figure 4j) [24]. The changes in structure of the micelle of DPD are similar to those of DPM, which can be explained by the same rule. However, the difference is that the spherical micelles formed by DPD have a hollow structure (Figure 5a), the rod-shaped micelles formed are longer than 200 nm (Figure 5b), and no liquid crystal appears under alkaline conditions. In addition, compared with acidic conditions, it can be seen that the spherical micelles formed by DPM and DPD tend to agglomerate under alkaline conditions to reach a stable state.

### 2.6. Foam Performance

**Figure 6:**
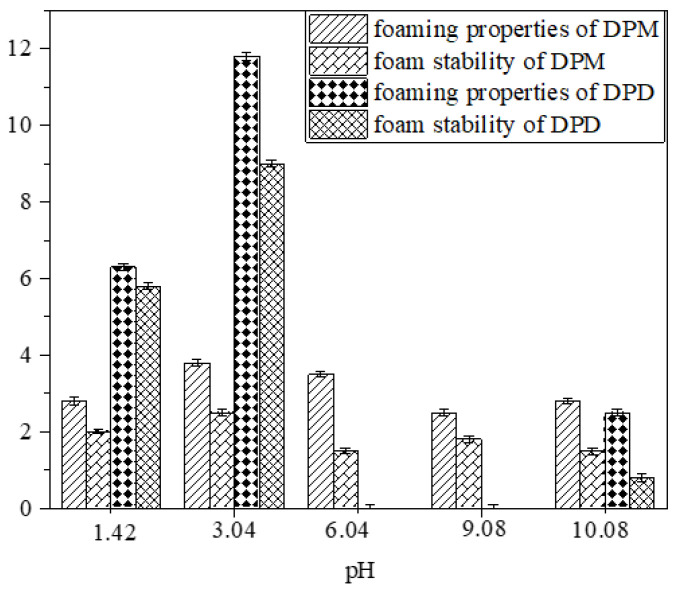
Foaming properties and foam stability.

The foam properties of DPM and DPD under different pH conditions are shown in Figure 6. The foaming performance and foam stability under acidic conditions are better than those under alkaline conditions. This may be because there are a large number of positive hydrogen ions under acidic conditions, which have a charge-attracting effect with the hydrophilic group phosphate negative ions in DPM and DPD. Therefore, the repulsion between the hydrophilic groups of the DPM and DPD molecules is weakened, and the foaming ability and foam stability are enhanced. Under alkaline conditions, OH^−^ and phosphate anions have a charge-repulsion effect, which strengthens the repulsion between the hydrophilic groups of DPM and DPD molecules, and their foaming ability and foam stability are relatively weaker than in acidic conditions [25]. When pH = 3.04, DPM and DPD had the strongest foaming ability and the best foam stability. In addition, the foam performance of diesters under acidic conditions is better than that of monoesters, because the molecular arrangements of diester surfactants are more similar to those of monoester surfactants. When pH = 6.04 and 9.06, the foaming capacities of DPD and DPM are poor, because the contents of H^+^ and OH^−^ in the solution are too low in the range of 6.04–9.06, and it is difficult to make DPD and DPM hydrophilic to form bubbles. As DPM has one more hydroxyl group than DPD, its hydrophilic property and foam performance are better than those of DPD. The foam heights of CTAB and SDBS reached 39 mL and 16 mL, respectively [11]. Moreover, the foaming performance of each was stable, and the foaming heights remained unchanged. By comparing the foaming properties of traditional surfactants CTAB and SDBS, it can be seen that the foaming properties of DPM and DPD are poor.

### 2.7. Emulsification Performance

Emulsifying performance is an important parameter used to characterize amphiphilic molecules. It could predict their applicability as emulsifiers in several applications, including shampoos, cosmetics, latex paints and the textile industry [26,27]. Phosphate ester surfactants have good affinity to the skin, and phosphorus-containing surfactants have strong emulsifying power and good stability. At the same time, rosin contains a three-membered phenanthrene ring structure, which has strong lipophilicity and good emulsifying properties.

**Figure 7:**
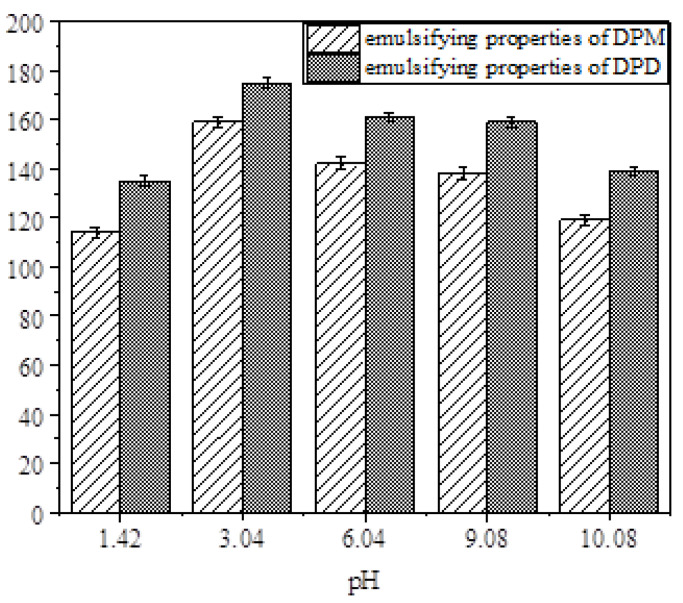
Emulsification properties.

The emulsification performances of DPD and DPM under different pH conditions are shown in Figure 7. It can be seen in Figure 7 that DPM and DPD perform best in emulsification under weakly acidic conditions (pH = 3.04). This may be due to the introduction of phosphoester groups. There are many positive hydrogen ions under acidic conditions, which have a charge-attracting effect on the hydrophilic groups of the negative phosphate ions in DPM and DPD. Therefore, the repulsion between the hydrophilic groups of the DPM and DPD molecules is weakened, and the emulsification performance is enhanced in both cases. In addition, the emulsifying power of the symmetric rosin-based phosphodiester surfactant DPD can reach 175 s, and its emulsifying performance is better than that of DPM surfactants. According to the number of hydrophobic tail chains, DPM and DPD can be divided into single-tail-chain and double-tail-chain surfactants. Under various pH conditions, the emulsification performances of the diesters are better than that of the monoester. This may be because DPD has two hydrophobic tail chains [28]. The emulsifying power values of DPM and DPD can reach 159 and 175 s, respectively. Compared with the emulsification times of 73 and 43 s for SDBS and CTAB [11], those emulsification times are relatively long. Compared with the emulsification times of 153 and 63 s for the rosin-based surfactants DDPDS and RETA, the two synthetic surfactants DPM and DPD have excellent emulsification performances [7,11].

### 2.8. Hydrophilic and Lipophilic Balance Value (HLB)

The HLB value is used to measure the hydrophilicity and lipophilicity of surfactants. The greater the HLB value, the stronger the hydrophilicity [29]. According to formula 3.3.3, the HLB values of DPM and DPD are 13.9 and 11.9, respectively. DPM has better hydrophilicity than DPD, which is caused by the difference between the rigid skeleton structure of the strongly lipophilic ternary phenanthrene ring and the weakly hydrophilic hydroxyl group in the molecular structure.

## 3. Experimental Section

### 3.1. Main Raw Materials, Reagents and Instruments

Dehydroabietic acid with 99% (HPLC) purity was purchased from Wuhan Xinxin Technology Co., Ltd., Wuhan, China. Oxalyl chloride: AR, McLean. N-methyldiethanolamine (MDEA): AR, McLean. Phosphorus pentoxide: AR, McLean. Calcium hydride: AR, McLean. Dichloromethane: AR, Bei Jing chemical plant. Ethyl acetate: AR, Bei Jing chemical plant. Deionized water was purchased from the Institute of Semiconductors, Chinese Academy of Sciences.

OSB2100 Rotary Evaporator; Nicolet FTIR 6700 Fourier Transform Infrared Spectrometer (The Thermo Nicolet Instrument Company, Waltham, MA, USA); Bruker AV III 400 MHz Nuclear Magnetic Resonance Spectrometer (Switzerland Bruker Company, Fällanden, Switzerland); JYW-200B automatic interface tension meter (Chengde Jinhe Instrument Manufacturing Co., Ltd., Chengde, China).

### 3.2. Synthesis Method

The synthesis of the target compound has a great relationship with the temperature and the molar ratio of the reactants. In order to obtain products with higher purity, the synthetic experimental conditions were set as shown in Table 1. The synthesis methods for intermediate products (rosin acid chloride) and I (DDAM) are shown in reference [12].

#### 3.2.1. Preparation of Rosin-Based Phosphorus Monoester (DPM)

Dissolve rosin monoester DDAM in tetrahydrofuran, add phosphorus pentoxide in a certain proportion, react at 60 °C for 6 h, add a small amount of deionized water and hydrolyze at 65 °C for 3.5 h. The solvent was removed by rotary evaporation. The crude product was dissolved in a mixture of water, isopropanol and n-hexane. The upper layer was separated by standing and dried overnight. The rotary evaporation yielded an acid orange-yellow transparent viscous compound (DPM) with a yield of 70.8%.

#### 3.2.2. Preparation of Rosin-Based Phosphodiester (DPD)

Dissolve the rosin monoester DDAM in tetrahydrofuran, add phosphorus pentoxide in a certain proportion, react at 60 °C for 6 h, add a small amount of deionized water and heat to 80 °C for hydrolysis for 2 h.

The solvent was removed by rotary evaporation; the crude product was dissolved in a mixture of water, isopropanol and n-hexane. It was left to stand to remove the lower layer and dried overnight with anhydrous magnesium sulfate. Finally, the solvent was removed by rotary evaporation to obtain an acid orange–yellow transparent viscous compound (DPD) with a yield of 80.3%.

### 3.3. Performance Test

#### 3.3.1. FTIR Measurement

FT-IR spectra were analyzed on a Nicolet FT-IR 6700 spec-tropometer (Madison, WI, USA) in dry air at room temperature. Using attenuated total reflection technology (ATR) analysis, we ground and pressed the dried compound with KBr and scanned the infrared reflectance in the interval of 500–4000 cm^−1^.

#### 3.3.2. NMR Analysis

^13^C NMR, ^31^P-NMR and ^1^H-NMR spectra were recorded on a Bruker AV III 400 MHz spectrometer at 25 °C in deuterated chloroform.

#### 3.3.3. Hydrophilic and Lipophilic Balance Value (HLB)

In this study, the group number method was used to calculate the HLB values of surfactants [30]. The group number method was used to decompose the entire molecular structure of the surfactant into a single group and calculate the contribution of each group to the HLB value. The calculation formula is as follows:

HLB = 7 + Σ Hydrophilic group−Σ Hydrophobic group.

#### 3.3.4. Other Measurements

The critical micelle concentration and surface tension were measured with the ring method. Emulsification, foam and TEM tests were the same as in reference [16].

The TEM test observes the morphologies and sizes of the surfactant aggregates. We configured the surfactants into aqueous solutions of different concentrations. One drop of the dilute aqueous solution was deposited on the surface of a carbon-coated copper grid and allowed to dry at room temperature for investigation by TEM.

## 4. Conclusions

Two decomposable surfactants, DPM and DPD, were synthesized. The analysis of FT-IR, ^13^C NMR and ^31^P NMR confirmed successful synthesis and optimized synthesis conditions. The γ_CMC_ value and CMCs of DPM and DPD are affected by pH, and the γ_CMC_ value under acidic conditions is smaller than that under alkaline conditions. When pH = 3.04, DPM and DPD are at their best in terms of foaming ability and foam stability. Under the conditions of pH = 1.42, 3.04, 6.04, 9.06 and 10.08, the emulsification performance of DPD was better than that of DPM. The feed ratio of n(P_2_O_5_)/n(DDAM) was 0.3333 in the synthesis, and the reaction temperature was 60 °C, which are the best reaction conditions for the synthesis of mono- and diesters. As pH increased, the morphologies of DPM and DPD micelles changed from spherical to rod-shaped to spherical, indicating that the aggregation states of the two surfactants are affected by pH.

## Figures and Tables

**Table 1:** Reaction conditions for the synthesis of rosin-based phosphorous ester.

Sample Number	P_2_O_5_ (mol)	DDAM (mol)	Temperature Reflex (°C)	Reaction Time (h)
1	1	3	60	6
2	1	3	70	6
3	1	3	80	6
4	3	1	70	6
5	3	1	60	6

**Table 2:** γ_CMC_ and CMCs of DPM and DPD at different pH values.

pH	1.42	3.04	6.04	9.06	10.08
γ_CMC_−DPM (mN·m^−1^)	37.782	39.57	40.987	44.21	43.104
CMC−DPM (mmol·L^−1^)	2.166	1.25	1.657	1.202	5.964
γ_CMC_−DPD (mN·m^−1^)	36.536	37.412	44.379	41.805	41.911
CMC−DPD (mmol·L^−1^)	0.631	0.498	0.612	0.199	0.321

## Data Availability

Not applicable.

## Supplementary Materials

The following supporting information can be downloaded at: https://www.mdpi.com/article/10.3390/molecules28073091/s1. Figure S1: ^13^C NMR of DPM. Figure S2: ^1^H NMR of DPM. Figure S3: ^13^C NMR of DPD. Figure S4: ^1^H NMR of DPD. Figure S5: ^31^P NMR of all samples. Figure S6: ^31^P NMR of sample 1 in alkaline conditions. Figure S7: ^31^P NMR of sample 5 in alkaline conditions.

## Sample Availability

Samples of the compounds DPM and DPD are available from the authors.
