# Supplementary material for: Design and Properties of Natural Rosin-Based Phosphoester Functional Surfactants
Source: Molecules. 2023 Mar 30;28(7):3091. doi: 10.3390/molecules28073091 (PMC10096330; doi:10.3390/molecules28073091)
Supplement: Supplementary file 1 [file molecules-28-03091-s001.zip › molecules-2298553-supplementary.pdf]

molecules

MDPI

Design and Properties of Natural Rosin-Based Phosphoester Functional Surfactants

Maogong Wang [1,†], Xiaofang Yang [2,†], Bing Han [2], Shifeng Zhang [2], Chunrui Han [2,*] and Changlei Xia [3]

[1] CNPC Engineering Technology R&D Company Limited, Beijing 102206, China
[2] MOE Engineering Research Center of Forestry Biomass Materials and Energy, Ministry of Education, Beijing Forestry University, Beijing 100083, China
[3] College of Materials Science and Engineering, Nanjing Forestry University, Nanjing 210037, China
* Correspondence: hanchunrui@bjfu.edu.cn
† These authors contributed equally to this work.

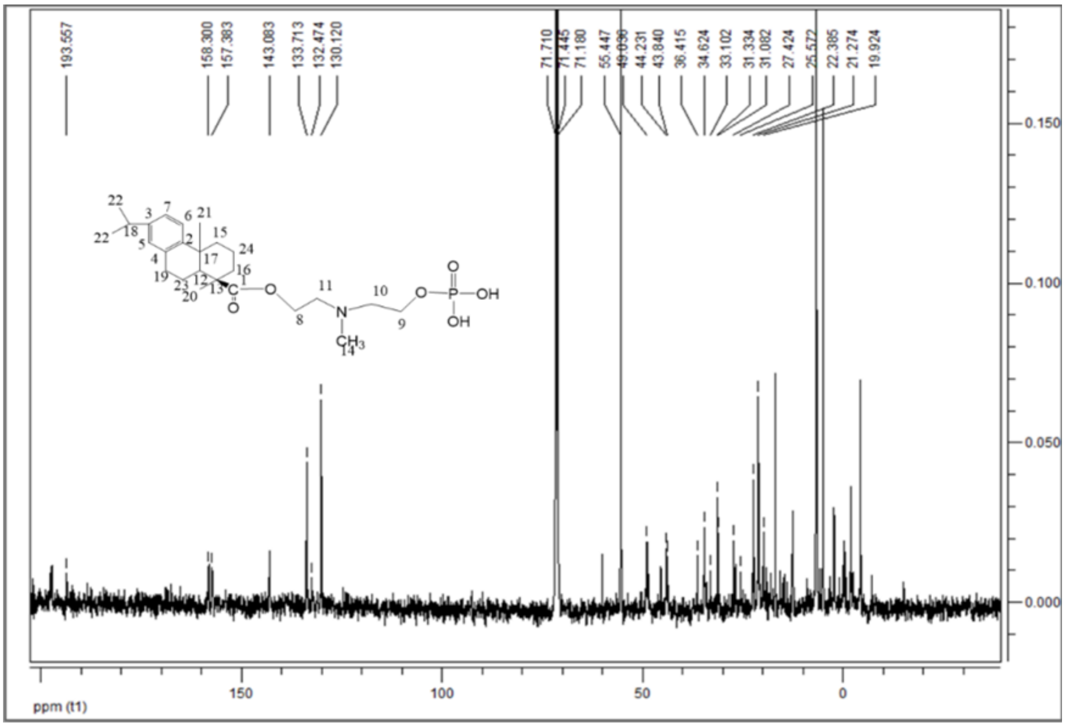

Figure S1. ^{13}C NMR of DPM.

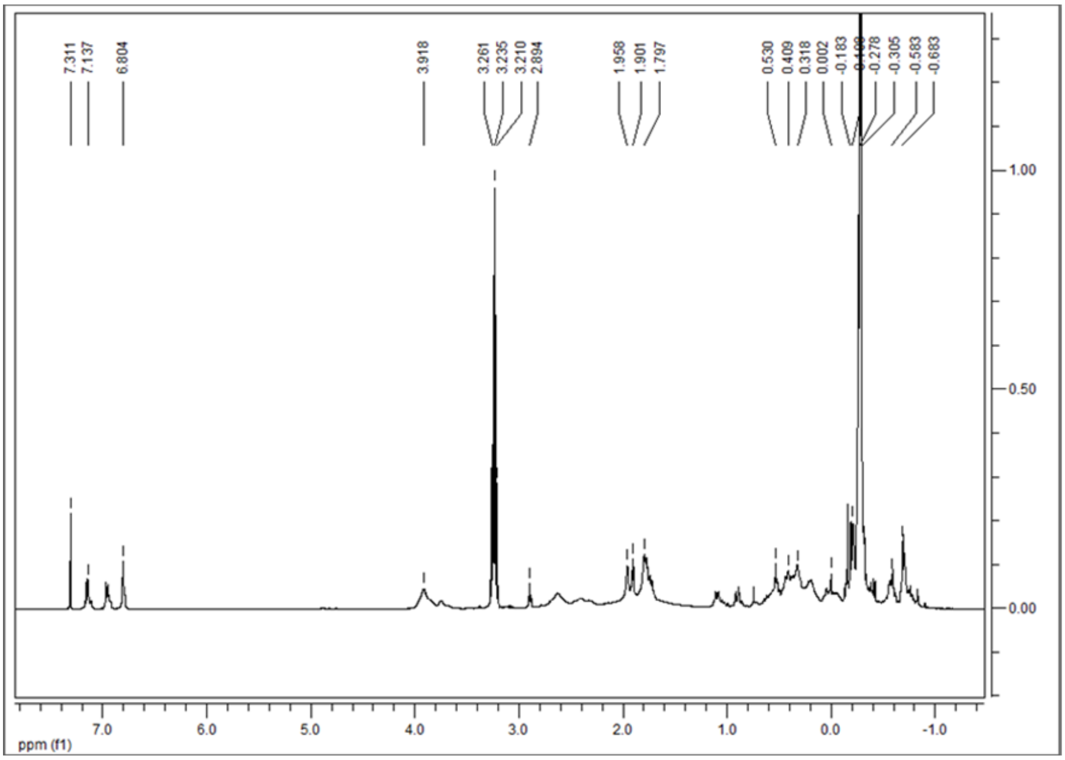

Figure S2. ^{1}H NMR of DPM.

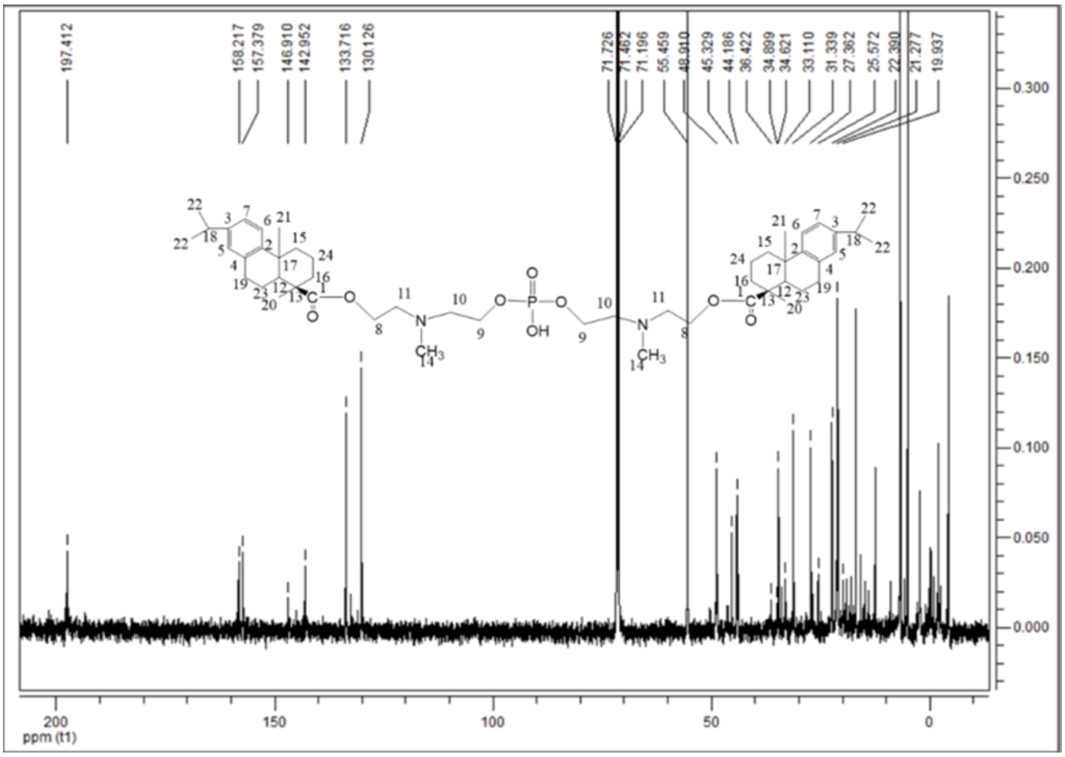

Figure S3. ^{13}C NMR of DPD.

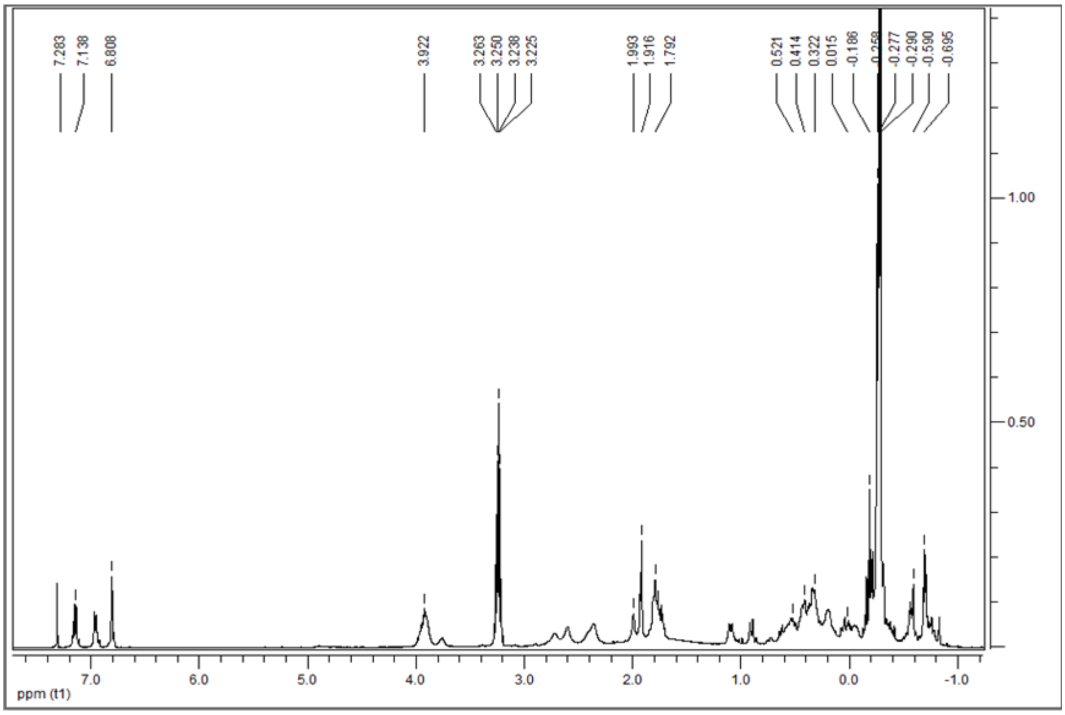

Figure S4. ^{1}H NMR of DPD.

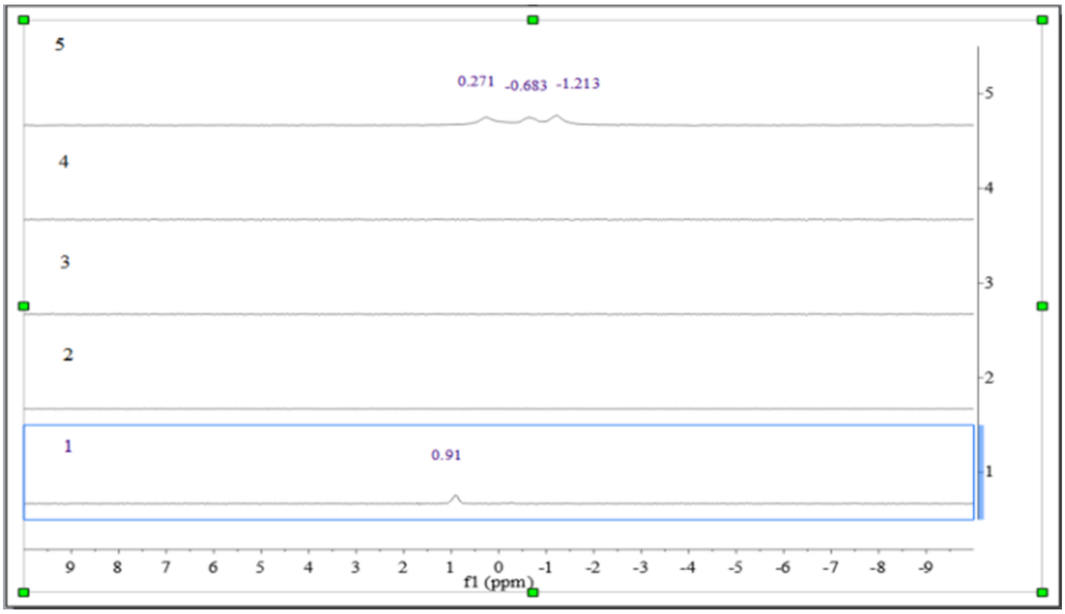

Figure S5. ^{31}P NMR of all samples.

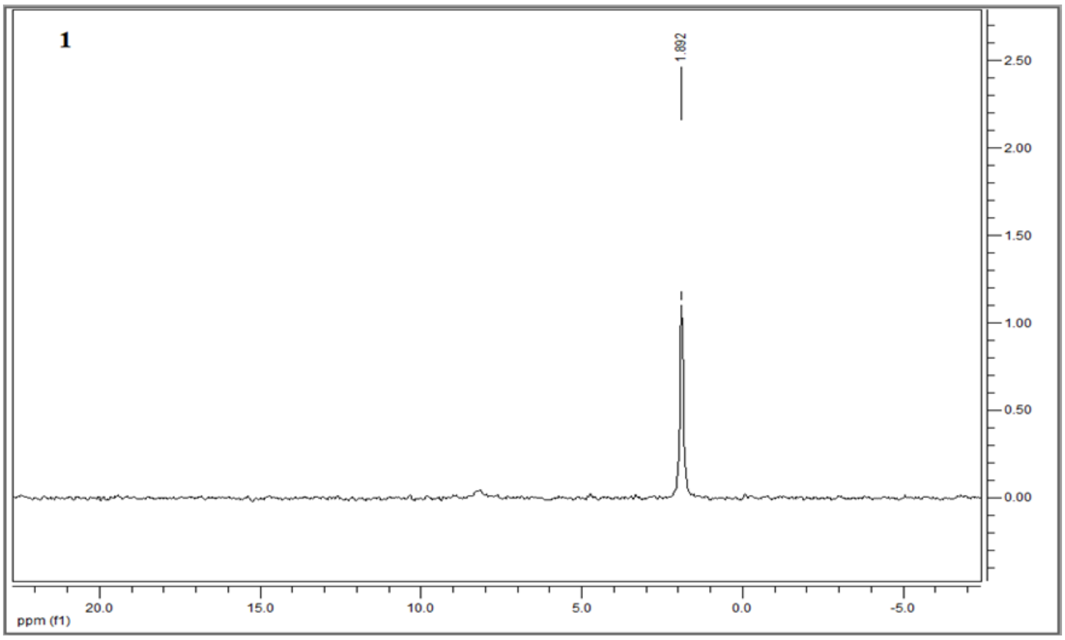

Figure S6. ^{31}P NMR of sample 1 at alkaline condition.

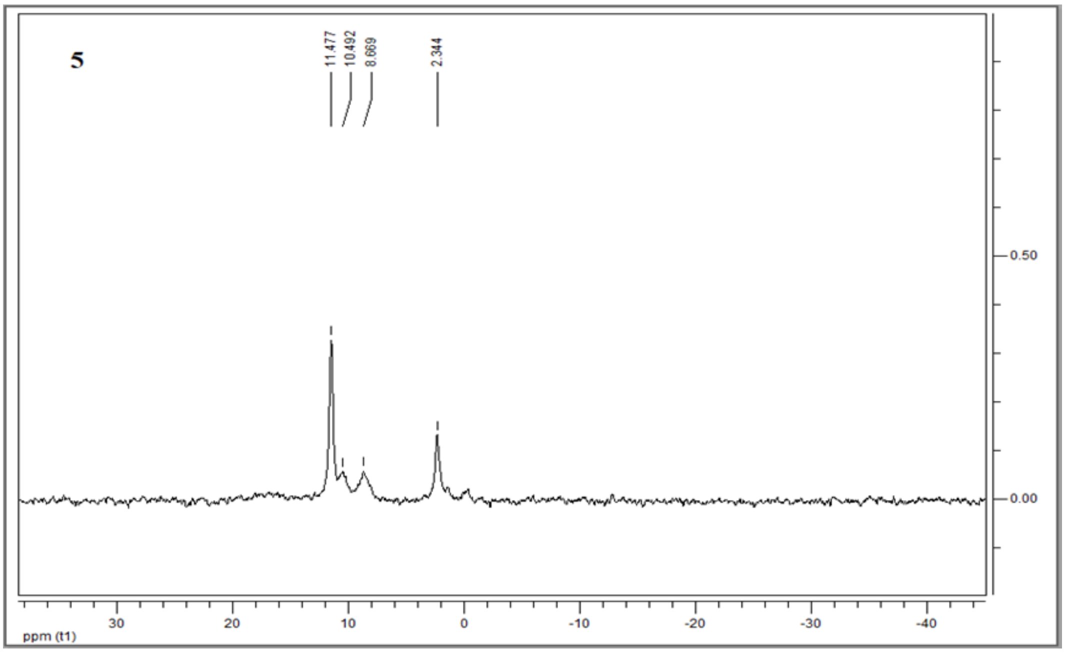

Figure S7. ^{31}P NMR of sample 5 at alkaline condition.
